# Observations on the Luxation of the Bones of the Pelvis

**Published:** 1790

**Authors:** M. Enaux

**Affiliations:** Professor of Midwifery, one of the Surgeons of the General Hospital, and Member of the Academy of Sciences at Dijon


VII. *Observations on the Luxation of the Bones of the Pelvis. By M.* Enaux, *Professor of Midwifery, one of the Surgeons of the General Hospital, and Member of the Academy of Sciences at Dijon.* — Vide *Nouveaux Memoires de l'Academie de Dijon, pour la Partie des Sciences et Arts,* Vol. III.

ALL the authors who have written on the diseases of the bones have treated very fully of fractures of the bones of the pelvis; but from their silence concerning the luxation

 of of thefe bones it would feem as if the firmnefs with which they are connected had led them to confider fuch an accident as impoffible. We have the greater reafon to prefume this, as the accidental feparation of the bones of the pelvis after difficult labours was for a long time a matter of difpute in the fchools. The luxation of thefe bones, produced by an external caufe, was till then little known; and as this difeafe has not hitherto been defcribed in books, M. Enaux has been induced to point out, in the paper before us, the marks by which it may be afcertained. It happens, however, he obferves, that he is able to give an account of only one cafe of this fort from his own practice; and as a fingle inftance can hardly be expected to afford all the fymptoms attendant on fuch accidents, he has thought it right to avail himfelf of two cafes of the fame kind, quoted by M. Louis in his Obfervations on the Separation of the Bones of the Pelvis. Both thefe cafes prove the poffibility of fuch a feparation from an external caufe.

The firft of thefe two cafes is from Baffius. The patient was a young man, much debilitated, who, while exerting himfelf violently in fencing, felt an acute pain at the part where the the os ilium is united to the os facrum. Baffius faw the patient on the third day after the accident, and convinced himfelf of the diflocation of the os facrum. Attempts were made to reduce it; but thefe proving ineffectual, Baffius contented himfelf with the ufe of ftrengthening applications, and the patient gradually recovered, and fuffered no inconvenience from the accident.

The fecond cafe is given on the authority of M. Philippe, who met with a luxation of the os ilium, occafioned by the fall of a fack of wheat on the right fide of the os facrum. The fymptoms at firft were fo flight, that the patient was able to go about his bufinefs for three days; but at the end of that time the pain became confiderable, and on the fifteenth day he applied to a furgeon, who bled him feveral times. The fymptoms, however, continued to become more alarming, and on the twenty-fifth day M. Philippe was confulted. The nature of the cafe was then carefully inquired into, but no luxation could be perceived of any of the bones of the fpine or pelvis. The tenfion of the belly, however, fufficiently indicated the hopelefs fituation of the patient, who died five days after. On diffection M. Philippe discovered a dislocation of the os ilium, which was nearly three inches distant from the os sacrum; together with a considerable quantity of purulent matter in the pelvis.

These two cases confirm the possibility of a luxation of the bones of the pelvis from an external cause; but the first of them our author thinks deserves rather to be considered as an instance of diastasis than of common luxation.

The case, he observes, related by M. Philippe is very different from the other. In this the cause of the dislocation was violent. A sack of wheat, of more than three hundred pounds weight, must, he thinks, have been very capable of separating the parts on which it fell, however strongly they might be united to each other. The contusion and laceration must have been proportioned to the violence excited, and the effects of these, from the consequent inflammation and suppuration, were (as actually happened) less likely to be felt immediately than some time after the accident. Thus we find, remarks our author, that the patient continued for some days to follow his business, but that suppuration began then to take place; and he is of opinion that it is to this suppuration, by which the substance of the contused contufed and lacerated ligaments was deftroyed, that we are to attribute the greatnefs of the fpace between the bones, rather than to the diflocation which took place at the time of the accident. The circumftances of both thefe cafes, he obferves, are effentially different from thofe of the following one, which occurred in his own practice; but he has thought a recital of the preceding facts neceffary for the purpofe of bringing together all the fymptoms likely to accompany and diftinguifh luxations of the bones of the pelvis.

The fubject of this cafe was a Tiler, at Dijon, aged thirty years, and of a robuft conftitution, who fell from a height of forty feet. He was immediately conveyed to the General Hofpital, where our author faw him foon after the accident. He found him in bed, complaining of very acute pain, which he defcribed as extending from the groin through the interior part of the pelvis to the facro-iliacal fymphyfis. The leg was in a ftate of flexion, with the foot turned outwards. The whole inner furface of the thigh was contufed.

The greater part of thefe appearances feemed to indicate a diflocation of the thigh bone, or rather a fracture of the neck of that bone.

M. Enaux

M. Enaux was the more confirmed in this opinion upon taking hold of the limb by the foot, and feeling the crepitation occafioned by the friction and inequality of the bony pieces. His colleague, M. Chauffier, who had undertaken to fupport the pelvis during the reduction, likewife felt this crepitus. The author obferves, however, that he did not long continue in this opinion concerning the nature of the difeafe; for in the fracture of the neck of the thigh bone the leg gradually returns to a ftate of flexion whenever the extenfion ceafes, but in this cafe no fuch flexion took place, although the leg was at liberty; on the contrary, the limb remained in the fituation in which it had been placed by a flight extenfion.

Our author now examined accurately the articulation of the thigh, and while he was doing this, the patient, by an involuntary motion, brought on the flexion of the leg, and he then was convinced there was no diflocation of the joint of the hip.

The crepitus had been too fenfibly felt to allow him to fuppofe there was no fracture; he therefore was led to examine the bones of the pelvis, and he found the os pubis of the left fide exceeding in height that of the right at leaft two two fingers breadth ; but the pain in the pelvis becoming more acute, and the patient being feized with fhivering, he thought it right to defer the reduction till another time. Occafional bleeding, a ftrict diet, clyfters, and a favourable pofition, were the means employed in the treatment.

It was not till the fourth day that he was able to proceed in his examination. The bones of the pubis were ftill in the fame ftate as before, and M. Enaux having attempted in vain to reduce them, was inclined to think there was a fracture of thefe bones, for, he candidly confeffes, he had not the leaft fufpicion of luxation. He difcovered this, he fays, only by accident. It was in bending the thigh, and bringing it towards the belly, (the leg being at the fame time alfo in a ftate of flexion) that the left os pubis defcended fo as to be on a level with the right ; but the pain at the facro-iliacal fymphyfis became fo acute, that he was foon obliged to alter this pofition of the thigh. Still, however, repeating the fame manœuvre, he placed one hand on the facro-iliacal fymphyfis, and another on the pubis, while he again bent the thigh. He now clearly perceived the motion communicated from one fymphyfis to the other by by each extremity of the bone; and at the ſame time the firmneſs of the tuberoſity of the iſchium, together with the evenneſs of the ſpine of the ilium, convinced him there was no fracture.

The pain the patient experienced obliging him to deſiſt for the preſent from his endeavours to reduce the diſlocated parts, he was deſirous of attempting it again after ſome interval of time; but this ſecond attempt, like the former one, having occaſioned acute pain at the ſacro-iliacal ſymphyſis, he thought it right to content himſelf with the means already employed, and to leave the reſt to nature.

Notwithſtanding the imprudent conduct of the patient, who got up too ſoon, and without the author's conſent, the left os pubis was brought down to within leſs than a finger's breadth of the right, and the patient quitted the hoſpital at the end of ſeven weeks, a little lame, but capable of following his buſineſs.

In ſome reflections on this caſe the author obſerves, that accidents of this kind muſt be ranked among the more rare and extraordinary events of ſurgery, and that a fracture of theſe bones muſt be more frequent than their diſlocation, becauſe the os ilium affording ſo great a ſurface, muſt, on that account, be more liable to become fractured. In the case just now related, he observes, the tuberosity of the ischium was required to support the whole effort from below upwards to raise up and luxate the ilium, and this could not be done without injury to all their articulations.

The ecchymosis on the inner part of the thigh, which appeared immediately after the accident, he ascribes to an extravasation within the pelvis. By describing all the appearances which led him at first to consider the accident as a fracture, he has endeavoured, he tells us, to put others on their guard against a similar error.

The three cases he has related afford, he thinks, sufficient proofs of a luxation of the bones of the pelvis from an external cause; and at the same time may serve to show that the reduction of the parts not being always possible, a palliative mode of treatment becomes the only one to which we ought to have recourse, lest we should expose the patient to more evident danger.

